# Graphene Reinforced Anticorrosion Transparent Conductive Composite Film Based on Ultra-Thin Ag Nanofilm

**DOI:** 10.3390/ma15144802

**Published:** 2022-07-09

**Authors:** Xiaowei Fan, Zenghua Zhao, Xiaoping Liang, Xuguo Huai, Chan Wang, Juncheng Liu, Chunyang Duan

**Affiliations:** 1 School of Material Science and Engineering, Tiangong University, Tianjin 300387, China; xiaowei_fan@126.com (X.F.); tjpulxp@tiangong.edu.cn (X.L.); huaixuguo@tiangong.edu.cn (X.H.); 2 School of Petrochemical Engineering, Liaoning Petrochemical University, Fushun 113001, China; zzh799@126.com (Z.Z.); wangchan0401@126.com (C.W.)

**Keywords:** transparent conductive film, ultra-thin Ag nanofilm, graphene, anti-corrosion, electrospraying

## Abstract

Transparent conductive films are widely used in electronic products and industrial fields. Ultra-thin Ag conductive nanofilm (ACF) was prepared on a soda lime silica glass (ordinary architectural glass) substrate with industrial magnetron sputtering equipment with AZO (Al_2_O_3_ doped ZnO) as the crystal bed and wetting layer. In order to improve the corrosion resistance and conductivity of the ACF, graphene nanosheets were modified on the surface of the ACF by electrospraying for the first time. The results show that this graphene modification could be carried out continuously on a meter scale. With the modification of the graphene layer, the corrosion rate of graphene-decorated ACF (G/ACF) can be reduced by 74.56%, and after 72 h of salt spray test, the conductivity of ACF samples without modification of graphene can be reduced by 34.1%, while the conductivity of G/ACF samples with modification of graphene can be reduced by only 6.5%. This work proves the potential of graphene modified ACF to prepare robust large-area transparent conductive film.

## 1. Introduction

Transparent conductive film is an important component of instrument panels, electronic touch screens, optoelectronic devices, modern wearable electronics, and other devices [1,2,3]. Because the conductive layer has the function of low radiation and high infrared reflection, it can reflect the infrared heat energy to a great extent, so that the glass modified by the transparent conductive film has very low absorptivity [4]. Therefore, it also has important applications in industrial commodities such as automobile glass and building glass curtain walls, and can be used as green building materials to reduce internal and external heat exchange, thus enhancing comfort energy conservation. Therefore, it is of great significance to develop methods for the large-scale preparation of transparent conductive films.

ITO (indium tin oxide) is a typical representative of current high-performance transparent conductive films because of its excellent optoelectronic properties [5]. However, due to its intrinsic semiconductor properties, ITO shows a slow temperature response and sensitivity to acidic and basic environments [5,6,7]. Meanwhile, because indium is a rare material, its source is rather limited.

In order to replace ITO, Al-doped ZnO films (AZO) [8], metal meshes [9,10], and graphene and its composites [11,12,13] have been investigated by many scientists. However, the low conductivity of conductive materials and the problem of eliminating grid diffraction moiré fringes restrict the development of these approaches. Theoretically, there is a contradiction between high conductivity and high light transmittance, that is, improving the conductivity of transparent conductive films requires the guarantee of materials with good conductivity and large thickness, but in doing so, the light transmittance of materials will usually be sacrificed due to the mechanisms of light reflection, absorption, and scattering. Therefore, the selection of appropriate conductive materials is an important prerequisite for the realization of high-performance transparent conductive films. Silver (Ag) is the metal with the highest conductivity in nature. Due to its high electron density, it has the potential to provide excellent conductivity, electromagnetic shielding performance, and low radiation performance. Wang et al. obtained MXene/silver nanowire hybrid transparent conductive films, which showed good light transmittance and conductivity [14]. Tan et al. also loaded Ag nanoparticles and nanowires on non-woven fabrics, cellulosic paper, foam, and other matrix materials to enhance their electromagnetic shielding performance [15,16,17]. Wu et al. prepared transparent conductive materials by inkjet printing with Ag nanowire ink [18]. The above work proves the potential of silver materials to prepare transparent conductive films. However, it is difficult to achieve large-scale, controllable, and standardized preparation with these conductive films based on silver nanoparticles and nanowires due to the problems of preparation and assembly, which lead to the difficulty of meeting the practical application, especially as bulk commodities. Therefore, the development of large-scale and standardized methods for preparing Ag conductive films is of great significance for the application of high-quality conductive films.

During the conventional film forming process, Ag atoms nucleate first, and then grow in the vertical and horizontal directions to form three-dimensional grains; in this way, only three-dimensional structure grain-like “islands” can be formed when the thickness of Ag film is 10–20 nm, with which it is difficult to form a continuous conductive silver film [19]. However, when the Ag film continues to thicken to form a continuous conductive film, the Ag film will far exceed the limit thickness of 20 nm, and its light transmittance will reduce greatly. In addition, due to the fact that the Ag layer is easily oxidized, it is necessary to introduce a passivation layer to protect the ultra-thin silver film. In order to obtain ultra-thin Ag film and realize the unity of good visible light transmittance and conductivity, the concept of a coating wetting layer was proposed [20]. Ge and Ni were applied as the wetting layer, and a multi-layer composite film structure was prepared with antireflective materials such as MoO_3_, ITO, and MgF_2_ to realize composite Ag film with high conductivity [21,22,23]. However, so far, the preparation of multilayer materials with complex structure is still limited to small-size experimental samples. At the same time, the increase of the number of functional layers in the composite film often damages the conductivity and optical performance of conductive films.

Herein, we prepared an ACF on a soda lime silica glass (ordinary architectural glass) substrate with industrial magnetron sputtering equipment with AZO as the crystal bed and wetting layer. In order to improve the corrosion resistance and conductivity of the ACF, graphene nanosheets were modified on the surface of ACF by electrospraying. Moreover, this graphene modification could be carried out continuously on a meter scale. After modification, the corrosion rate of the ACF decreased by nearly an order of magnitude, which improved its durability significantly. Meanwhile, the ACF kept a low surface resistance of 11.1 Ω/sq and relatively high transmittance of 57.25% (weighted average of visible region). The method may also be used in flexible substrates such as polyethylene terephthalate to realize the preparation of large-area flexible transparent conductive films.

## 2. Materials and Methods

### 2.1. Preparation of Ultra-Thin Ag Conductive Film

Ultra-thin Ag conductive films were prepared by an industry magnetron sputtering system (VA-3348, Von Arddenne, Dresden, Germany) with the soda lime silica glass (ordinary architectural glass) as model substrates. After the cleaning of the substrate surfaces, SiN_x_, AZO, Ag, NiCr, and SiN_x_ layers were sequentially sputtered from Si, Al-ZnO, Ag, NiCr, and Si sputtering targets under the N_2_, O_2_-Ar, Ar, Ar, and N_2_ atmosphere, respectively. The initial vacuum of the sputtering chamber was 1 × 10^−6^ mbar, and the working pressure was maintained at 5 × 10^−4^ mbar. The SiN_x_ layer was deposited with reactive sputtering, in which a Si target was sputtered by a medium frequency AC power supply, 34 kHz, 600 V, 80 A, and N_2_ was the reaction gas, with the volume flow ratio of N_2_: Ar = 40:60. The AZO layer deposition also used a medium frequency AC power supply, 24 kHz, 500 V, 10 A; the molar ratio of Zn: Al in the target was 95:5; and the working gas included O_2_ and Ar, of which the volume flow ratio was 5:95. Both Ag and NiCr layers were deposited with a DC power supply, 300–500 V, 5–8 A, and the working gas was Ar. The thickness for every layer was monitored with a digital thickness monitor connected with a dual quartz crystal microbalance inside the sputtering chamber. The thicknesses of SiN_x_, AZO, NiCr, and SiN_x_ layers were 25 nm, 5 nm, 5 nm, and 48 nm, respectively. In order to study the effect of silver nanolayer thickness, we prepared intermediate ultra-thin silver nanolayer samples with the thickness of 3 nm, 6 nm, 9 nm, 12 nm, and 15 nm, respectively. The SiN_x_ layer improves the surface smoothness of the soda lime silica glass, prevents the precipitation of sodium ions in the glass, and improves the surface activity of the glass to facilitate the adhesion of the AZO layer. As a wetting layer and crystal seed, the AZO layer can inhibit the island effect of Ag sputtering deposition and reduce the critical thickness and surface roughness of continuous Ag film. The NiCr layer and outer SiN_x_ layer are protective layers to reduce the oxidation and vulcanization of Ag layer in atmospheric environment. The outer SiN_x_ layer is also an antireflection layer to improve the transmittance of visible light.

### 2.2. Preparation of Graphene-Decorated Ultrathin Ag Conductive Film (G/ACF)

Graphene nanosheets were prepared by direct exfoliation of vermicular graphite particles dispersed in deionized water without the presence of a surfactant. Then, the as-prepared graphene nanosheets were dispersed in the deionized water with a volume ratio of 5% ethanol to form a suspension with a concentration of 20 ppm. A conventional electrospinning setup was refitted by increasing the single nozzle of electrospinning to 5 nozzles in a row (nozzle spacing was 2 cm) for electrostatic spraying experiments. Meanwhile, a stepping motor was used to control the moving speed and range of the nozzles (50 mm/min over a width of 100 mm), so as to realize the uniform spraying of the graphene nanosheets on the substrates surface. The conductive glass substrates were placed on the rotating table with a diameter of 10 cm to receive graphene nanosheet microdroplets. During electrospraying, graphene nanosheet suspension was injected through 5 nozzles with the rate of the 10 mL/h for each syringe, with the electrospraying voltage as 15 kV (+10 kV for the nozzles, −5 kV for the G/ACF substrates). The distance between the nozzle tip and the G/ACF substrate was kept as 5 cm. After electrospraying, the excess amount of water on the G/CAF was wiped away with filter paper. The thickness of the graphene-decorated films was adjusted by the electrospraying time.

### 2.3. Characterization

The transparency and reflectivity of ultra-thin Ag conductive films and G/ACF films were measured using UV-Vis spectroscopy (UV-Vis spectrometer, PE Lambda 950, PerkinElmer, Waltham, MA, USA). The sheet resistance (Rs) was measured using a surface resistance meter (SRM-12TH, NAGY, Bavaria, Germany). The morphology of graphene nanosheets and the ACF and G/ACF films after corrosion was evaluated with a field-emission scanning electron microscope (FE-SEM, S-4800, Hitachi, Tokyo, Japan). The salt spray test was carried out in the salt spray box (PS-60, Beijing Yashilin testing equipment, Beijing, China) and the corrosion electrochemical experiment was tested by electrochemical workstation (CHI 660E, Chinstruments, Shanghai, China). Raman spectra and mapping (Renishaw, New Mills, Wotton-under-Edge, Gloucestershire, United Kingdom), microscopic characterization (DM2700M, Leica, Ernst-Leitz-Strasse 17–37 Wetzlar, Germany), and a contact angle test (CA500, Dataphysics, Stuttgart, Germany) were also carried out. In order to determine the chemical state of the silver layer surface, we prepared “Glass/SiN_x_/AZO/Ag/AZOII” samples, of which the AZOII layer was just used to prevent the oxidation of the silver. Its thickness was only about 3 nm, so that we could use an ordinary X-ray photoelectron spectroscopy (XPS, K-Alpha, Thermo Fisher, Waltham, MA, USA) to detect the silver layer surface.

## 3. Results & Discussion

### 3.1. Analysis and Investigation on ACF

**Figure 1:**
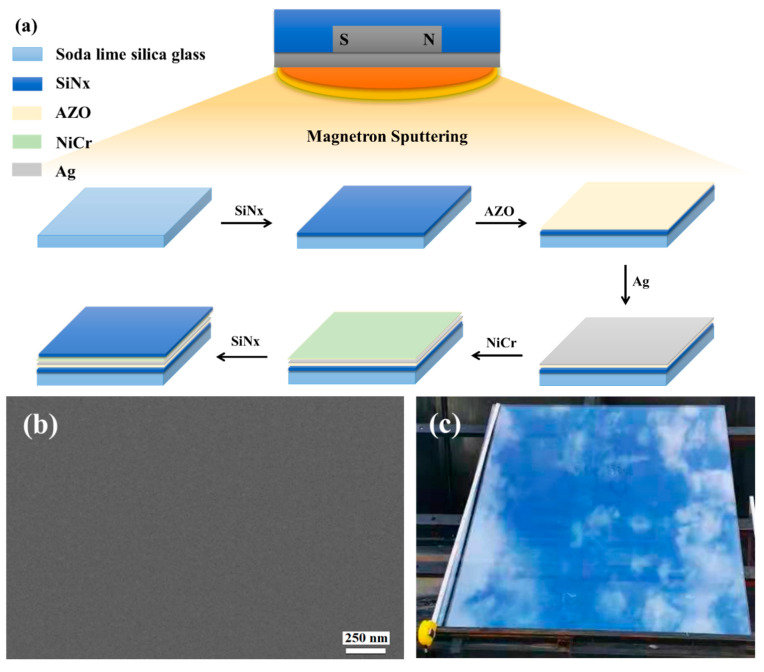

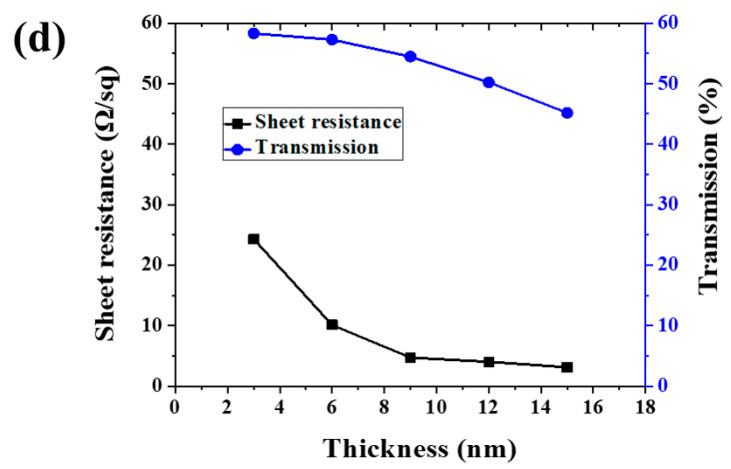
(**a**) Scheme of ultra-thin Ag conductive film (ACF) preparation procedure. (**b**) SEM image of ACF. (**c**) Optical photo of large-scale ACF samples deposited on the glass surface. (**d**) Sheet resistance and transmission of ACF with different thicknesses.

Figure 1a shows the scheme of the ultra-thin Ag conductive film preparation procedure, images, sheet resistance, and transmission.

Figure 1b is a SEM image of ACF prepared by magnetron sputtering. It can be seen that the ACF presents a flat surface and can show a clear morphology under the electron microscope without gold or carbon spraying modification, which proves the excellent conductivity of ACF. Figure 1c shows a typical optical photo of a large-area transparent conductive film deposited on the glass surface. The area of the conductive film was bigger than 1 m × 1 m, which demonstrates that the method provided in this paper has the ability to prepare a large-area transparent conductive film. Since the photo was taken on a dark background, the photo of the conductive glass shows the reflection of sky. The relationship between Ag layer thickness and ACF optical and electrical properties is shown as Figure 1d. As is shown in Figure 1d, with the increase of Ag nanolayer thickness, the surface resistance of ACF decreases sharply, that is, the conductivity is greatly improved, and its transmittance also decreases with the increase of Ag layer thickness. Specifically, the sample with a thickness of 3 nm had the largest surface resistance (24.3 Ω/sq), that is, the lowest conductivity and the highest transmittance (58.29%). The poor conductivity may have been caused by the incomplete film formation of the Ag nanofilm. With the increase of Ag nanofilm thickness, the film-forming is improved, and the conductivity of ACF is also improved. When the thickness was increased to 15 nm, the surface resistance was 88% lower than that of the 3 nm sample. However, the optical properties gradually decrease with the increase of Ag film thickness. The weighted transmittance of the visible region of the sample with a thickness of 3 nm was 58.29%, but when the thickness of the Ag nanofilm increased to 12 nm, the transmittance decreased significantly. When the thickness increased to 15 nm, the transmittance decreased to 77.46% of the sample with a thickness of 3 nm. The above results prove the contradiction between the conductivity and light transmittance of Ag nanofilm. Through the trade-off of these two properties, samples with a thickness of 6 nm and 9 nm had the most potential to prepare large-scale transparent conductive films; therefore, we selected 6 nm samples (marked as Sample 1) and 9 nm samples (marked as Sample 2) as the research objects.

**Figure 2:**
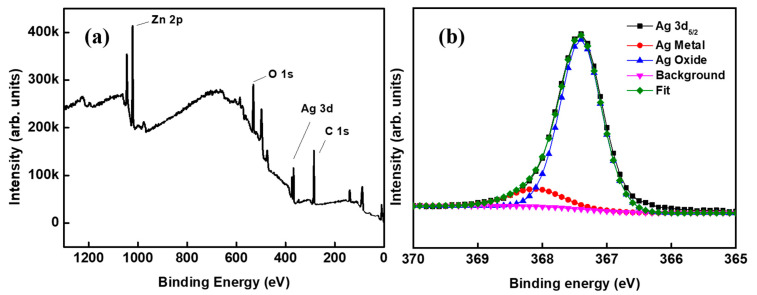
XPS spectra of the sample: (**a**) the full spectrum and (**b**) Ag 3d5/2 peak fitting spectrum.

In a multilayer composite film, the interfacial stability between any two layers needs much attention. Herein, the state of interface between the metal Ag layer and oxide AZO layer in the ACF film is worthy of special research. The XPS full spectrum and Ag 3d5/2 peak fitting spectrum are shown as Figure 2a, Figure 2b, respectively. As can be seen from Figure 2a, the main chemical elements in the sample were zinc, oxygen, silver, and carbon. Carbon can come from the adhesion of organic matter on the sample surface due to the contact between the sample surface and the plastic sample bag. The binding energy peak of Ag 3d5/2 was at 367.3 eV, which is 0.8 eV to the right of the reported position at 368.1 eV [24]. The shift of this position was likely mainly due to the slight oxidation of Ag [25]. After the peak separation and fitting, the Ag 3d5/2 peak was divided into two peaks: the peak of metal Ag and the peak of AgOx oxide, in which x should take an insignificant value. This shows that the Ag layer is a chemical state in which Ag metal and Ag oxide coexist. Terry et al. observed similar phenomena at the MgO/Ag interface, and thought that the existence of the AgOx peak may be due to the migration of charges or ions at the interface [26].

### 3.2. Analysis and Investigation of G/ACF

**Figure 3:**
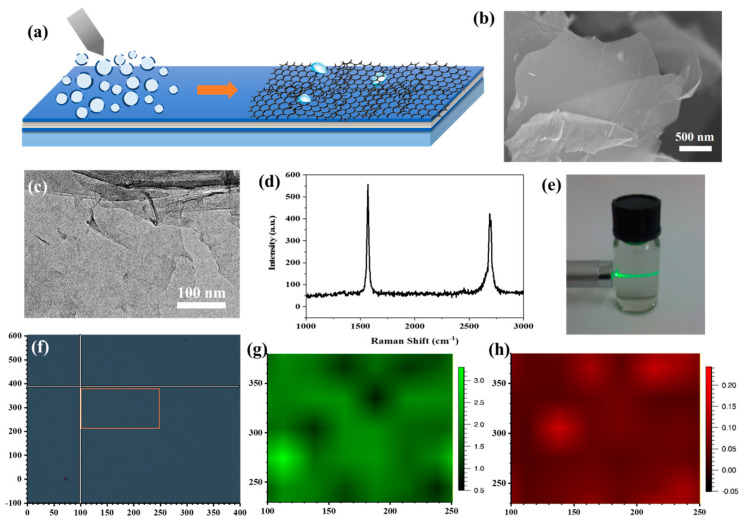
(**a**) Scheme of electrospraying for G/ACF film preparation procedure. (**b**) SEM image of graphene nanosheets. (**c**) TEM image of graphene nanosheets. (**d**) Raman spectra of graphene nanosheets. (**e**) Tyndall effect of graphene nanosheets suspension. (**f**) Image of the Raman mapping area. (**g**,**h**) Raman mapping results of G/ACF film.

Graphene, which is composed of a densely packed sp2-honeycomb network of C, shows excellent chemical inertness and impermeability to molecules even as small as He, thus enabling it protect the underlying metal substrates from corrosion [27]. Liquid exfoliation is a facile and high-yield approach to produce pristine few-layer graphene nanosheets with low lattice defects and the potential to provide high anti-corrosion ability [28]. Figure 3a shows the scheme of electrospraying for the graphene film preparation procedure on ACF. Electrospraying is a novel film-forming technology which facilitates nanostructures to assemble into uniform films under a strong electric field [29].

The homogeneous two-dimensional graphene nanosheets were firstly prepared by direct exfoliation. The continuity and wrinkles in the graphene nanosheets are obviously shown in the SEM (Figure 3b) and TEM (Figure 3c) images, which show no obvious fractures. Raman responses were collected by a Renishaw micro-Raman system with a 532 nm wavelength laser and a 100× magnifying lens from the as-prepared graphene nanosheets to confirm their quality. Raman results (Figure 3d) obtained on the graphene nanosheets show a negligible D peak (~1340 cm^−1^) and a sharp G peak (~1548 cm^−1^) and 2D peak (~2680 cm^−1^), which confirmed the high quality of the as-prepared graphene nanosheets together with the morphology measurements [30]. Figure 3e shows the Tyndall effect of the graphene nanosheet suspension in Milli-Q water with a concentration of 20 ppm, which proves the uniformity of the suspension. The suspension was then injected into the syringes of the home-built electrospraying setup and sprayed through five nozzles with the rate of the 10 mL/h onto the surface of the ACF. The ACF modified glass substrates were placed on the rotating table with a diameter of 10 cm to receive graphene nanosheet microdroplets. Under the action of high voltage and micro nozzle, the suspension is evenly adsorbed on the ACF surface in the form of microdroplets, and the graphene nanosheets form a uniform film with the rupture of microdroplets. Moreover, the thickness of the graphene decoration films was adjusted by the electrospraying time. In order to evaluate the quality of the graphene films, Raman mapping was conducted on the surface of a typical graphene-decorated ACF (G/ACF) sample (electrospraying time = 20 min) with the area of 150 μm × 150 μm (Figure 3f). The ratio of the G to 2D peak intensities (IG/I2D) and D to 2D peak intensities (ID/I2D) are used to evaluate the thickness, defects, and uniformity of the graphene films [31]. As shown in Figure 3g, the IG/I2D indicates good uniformity in the Raman mapping test range, and multilayered graphene films were formed on the surface of the ACF. Moreover, the low value of the ID/I2D shown in Figure 3h demonstrates that the as-prepared graphene films had high quality and few defects. Raman results indicate that a complete graphene film can be formed on the ACF surface after 20 min of electrospraying experiment. Therefore, we carried out electrospraying experiments on Sample 1 and Sample 2 surfaces for 20 min and 30 min, respectively, and marked the graphene-decorated samples as Sample 1G20, Sample 2G20 (20 min), and Sample 1G30 and Sample 2G30 (30 min).

### 3.3. Effect of Graphene on Optical and Surface Resistance

**Figure 4:**
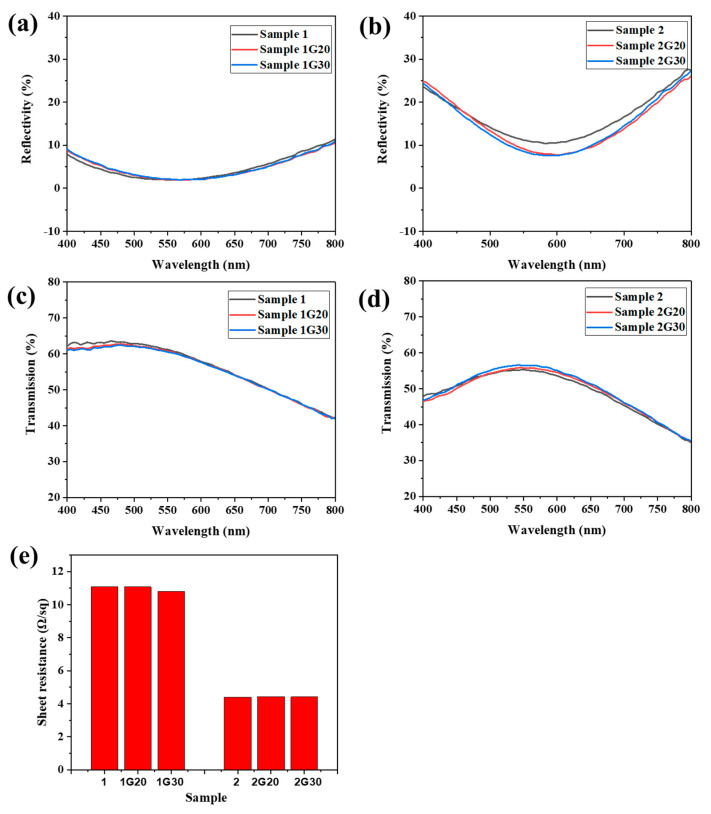
(**a**–**d**) Reflection and transmission spectra of ACF and G/ACF samples. (**e**) Sheet resistance of ACF and G/ACF samples.

Figure 4a–d show the reflection and transmission spectra of ACF samples with and without graphene decoration. Due to the thinner Ag film, Sample 1 showed lower reflectivity and higher transmissivity in the range of 400 nm to 800 nm compared with that of Sample 2. Meanwhile, because the thickness of the modified graphene film was thin and uniform, there was no significant change in the reflection and transmission properties of the graphene modified samples. The reflectivity of Sample 2G20 and 2G30 even dropped ~3% in the range of 520 nm to 700 nm compared to their unmodified counterpart, which might be attributed to the change of the incident path of light and partial absorption of reflected light by the graphene layer.

Figure 4e shows the sheet resistance of the ACF samples with and without graphene decoration. Sample 1 showed a much higher sheet resistance compared to Sample 2, which is consistent with the difference of Ag layer thickness between the two samples. When the graphene layer was modified, all the modified samples showed a surface resistance similar to that of the original samples. Although the perfect monolayer graphene has a much higher conductivity than metal in theory, it is difficult to have graphene nanosheets show excellent electrical properties similar to metal due to the influence of the preparation process, layered stacking, boundary effect, and other factors [32]. However, in this work, because Ag nanofilms have excellent conductivity and the graphene film attached to their surface form a parallel relationship with the substrate, the overall conductivity of the G/ACF samples was not greatly affected. The above results indicate that the modified graphene layer will not have a bad effect on the optical and electrical properties of the original sample, and the difference between the optical and electrical properties of Sample 1 and Sample 2 proves the contradictory relationship between conductivity and light transmittance. However, an ACF with excellent conductivity is easily oxidized and corroded, resulting in reduced conductivity. If the modified graphene layer can protect it without affecting its optical and electrical properties, the comprehensive properties of the transparent conductive film will be further improved.

### 3.4. Corrosion Resistance of G/ACF

**Figure 5:**
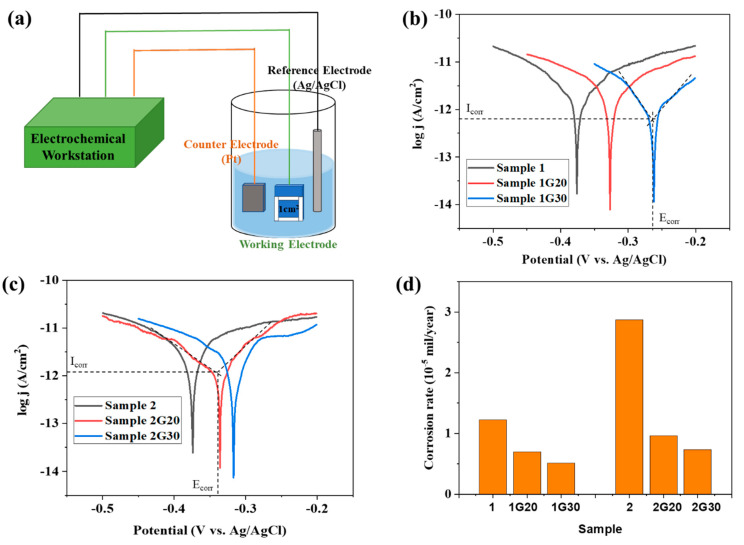
(**a**) Scheme of corrosion electrochemical test diagram of ACF and G/ACF samples. (**b**,**c**) Tafel plots of ACF and G/ACF samples. (**d**) Corrosion rates of ACF and G/ACF samples.

The electrochemical method is considered to be a reliable method to evaluate the corrosion resistance of the protective layer [33]. Figure 5a shows the scheme of the corrosion electrochemical test diagram of the ACF samples with and without graphene decoration, where the size of the electrochemical test sample is 10 mm × 10 mm, cut from the large sample (about 150 mm × 150 mm). A three-electrode system consisted of the samples to be tested with an exposed area of 1.0 cm^2^ as the working electrode, Ag/AgCl electrode as the reference electrode, and a Pt sheet as the counter electrode. Silicone rubber was used to expose the working electrodes with the desired area, and all measurements were conducted in 5% NaCl solution. The CHI 660E electrochemical workstation was used to provide the applied potential and to record data.

Figure 5b,c show the Tafel analysis results of all samples, in which the corrosion current density (Icorr) and the corrosion potential (Ecorr) could be determined by the tangent lines of the polarization curves of each sample. It can be seen that, under the scan rate of 5 mVs^−1^, all samples showed typical Tafel polarization. Compared with the bare ACF samples (Sample 1 and 2), the graphene-decorated ACF samples (Sample 1G20, 1G30, 2G20, and 2G30) all displayed a decrease in corrosion current density (Icorr). Meanwhile, the corrosion potential (Ecorr) of all the graphene-decorated ACF samples was shifted to a more positive potential. The positively shifting Ecorr means that the G/ACF samples can only be corroded at a higher potential compared with their bare counterparts, which indicates that the graphene layer can provide protection to the underlying ACF. The experimental result in Figure 5b,c also shows that the corrosion potentials of Sample 1 and 2 were almost the same, and the corrosion current of Sample 1 was much smaller than that of Sample 2. The consistence in Ecorr indicates that because they were prepared from the same material, even if they have different thicknesses, these two ACFs have the same possibility of corrosion in thermodynamics. Meanwhile, Sample 1 showed a smaller corrosion current than Sample 2 since Sample 1 has a thinner Ag film layer and a larger sheet resistance.

Corrosion rate is an important indicator of corrosion resistance and can be calculated using the *I_corr_* (extracted from the Tafel plots) by the following formula [27,28,29]:

(1) $$\;\;\;\;\;\;\;\;\;\;\;\;\;\;\;\;\;\;\;\;\;\;\;Corrosion\;rate=\frac{I_{corr}\times K\times E_{w}\;}{\rho A}$$

where the corrosion rate constant *K* is 3272 mm yr^−1^, the equivalent weight *E_W_* is 107.87 g for Ag, the material density *ρ* is 10.49 g/cm^3^, and *A* is 1 cm^2^ as the exposed area of the samples.

It can be seen that the corrosion rate of Sample 2 was significantly higher than that of Sample 1, which may be due to the thicker Ag layer and lower sheet resistance of Sample 2 (Figure 4d). After modifying the graphene, the corrosion rate of the G/ACF samples decreased significantly. Specifically, after modifying the graphene with different layer thickness, compared with the corrosion rates of Sample 1, Sample 1G20 and Sample 1G30 decreased by 42.62% and 58.2%, respectively. Meanwhile, compared with Sample 2, the corrosion rates of Sample 2G20 and Sample 2G30 decreased by 66.55% and 74.56%, respectively. The results show that for different samples, the decrease of corrosion rate is greater with the increase of graphene thickness. As the stacked graphene layer thickens, it can be more effective in blocking the pathways for dissolved oxygen and chloride ion intrusion through boundaries of graphene nanosheets to the underlying ACF surface, thus improving the anti-corrosion ability [34]. At the same time, due to the large corrosion rate of Sample 2 itself, graphene has a more obvious protective effect. When the surface of Sample 2 was protected by graphene with a thicker passivation layer, the corrosion rate was lower than that of Sample 1 and close to that of Sample 1 modified with graphene, which demonstrates that the graphene protection layer can protect ACF from corrosion.

Salt spray tests were used to evaluate the anti-corrosion ability of the ACF and G/ACF samples by providing artificial simulated corrosive environmental conditions. In this work, ACF and G/ACF samples with the size as 10 cm × 10 cm were used as the substrates for the evaluation of their anti-corrosion ability in a salt spray box (PS-60). During the tests, the samples were exposed to a seawater analogue that was prepared by 5% NaCl solution adjusted to pH = 6.5–7.2 with HCl under the ISO 3768-1976 method. Moreover, the samples were tested with microscope, SEM, optical, and electrical spectra after a certain time of salt spray.

Table 1 shows the microscope photos of all ACF samples with and without graphene decoration after salt spray test at different times. It can be seen from the micrograph that all the samples’ surfaces were clean and flat before the salt spray test. Under the same test conditions, the color of the graphene modified samples was darker than that of their unmodified counterparts. It can be seen that after 24 h salt spray test, bright spots representing corrosion points appeared on the sample surface, and all samples showed signs of corrosion. Especially for the unmodified graphene samples, the size of the etch point was larger and there were signs of linking into pieces. After 72 h of salt spray test, continuous corrosion areas with a size greater than 20 μm appeared on the surfaces of Sample 1 and Sample 2, while the corrosion spots on the surface of the sample modified with graphene film were sparse and within 10 μm.

**Figure 6:**
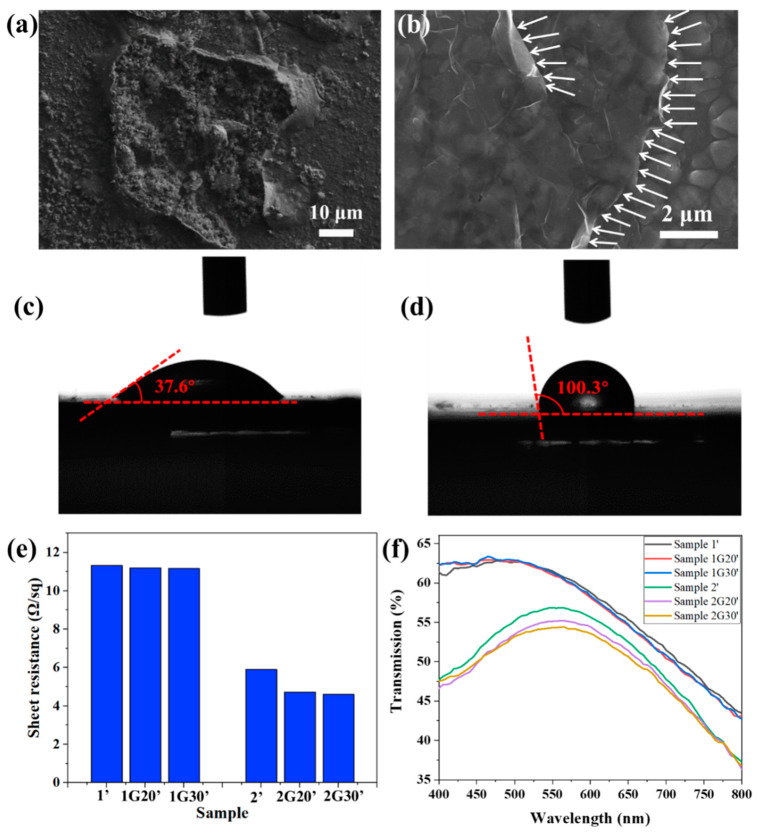
(**a**,**b**) SEM images of ACF (**a**) and G/ACF (**b**) samples after salt spray test. (**c**,**d**) Contact angles of the surface of ACF (**a**) and G/ACF (**b**) samples. (**e**,**f**) Surface resistance (**e**) and transmission (**f**) of ACF samples and G/ACF samples after 72 h salt spray test.

Figure 6a shows that after salt spray corrosion, a rough corrosion pit was formed on the surface of Sample 1, and its surface layer peeled off, indicating that the ACF was seriously damaged. Figure 6b shows that the surface of Sample 1G20, which was composed of micron particles, was still relatively complete after salt spray corrosion. There were grooves caused by corrosion between the particles, which were no longer as smooth and flat as shown in Figure 1b. The relatively complete edge of the graphene nanosheets can be seen clearly in the figure, as indicated by the white arrows in the figure, which shows that the graphene had stronger corrosion resistance. The difference of corrosion damage degree between Samples 1 and 1G20 is mainly due to the increase of corrosion potential, as shown in Figure 5b, and the decrease of corrosion rate, as shown in Figure 5d. Chai [35] transferred highly impermeable and transparent monolayer graphene onto the surface of Ag thin films as an ultra-thin protection barrier, revealing a high corrosion resistance to gases and liquids.

It has been reported that graphene possesses a low surface energy that leads to inherent hydrophobicity [36]. As shown in Figure 6c,d, the water contact-angle of the bare ACF substrate was 37.6°, and it rose to 100.3° after the decoration of graphene protective films. The increase in water contact angle reduces the contact area between the corrosion solution and the AFC surface and makes it less likely to accumulate on the AFC surface, thus reducing its corrosion effect on the AFC surface [37]. Figure 6e shows the surface resistance of these. The samples after salt spray test are distinguished by marking “ ’ ”, that is, they are marked as Sample 1’, Sample 1G20’, etc. After salt spray corrosion, the surface resistance of the samples all increased compared with that before corrosion, which shows that the corrosion process will indeed affect the conductivity of the conductive layer. Specifically, for Sample 1, Sample 1G20, and Sample 1G30 with a large surface resistance and strong corrosion resistance, the increase of surface resistance was less than 3% after the salt spray test, and the samples modified with graphene layers showed smaller surface resistance. For Sample 2, Sample 2G20, and Sample 2G30 with a small surface resistance and weak corrosion resistance, the increase of surface resistance was about 34.1%, 6.5%, and 4.3%, respectively. The sharp increase of surface resistance shows that the ACF film was corroded seriously, and the protection of the graphene layer greatly alleviated the deterioration of its conductivity, which is more obvious for samples with good conductivity. Figure 6f shows the transmission spectra of the ACF and G/ACF samples after corrosion. Compared to the samples before corrosion, the transmittance of Sample 1’, Sample 1G20’, and Sample 1G30’ did not change greatly, which is consistent with the change of surface resistance. For Sample 2 with a poor corrosion resistance, the transmittance was greatly increased due to the destruction of the Ag nanofilm, while Sample 2G20’ and Sample 2G30’ modified with graphene layers maintained the transmission characteristics from before corrosion, which further proves the protective effect of graphene layers on ACF.

## 4. Conclusions

In summary, we realized the preparation of large-area and high-quality ACF on soda lime silica glass by introducing an isolation layer and wetting layer in this work. Aiming at the problem that Ag nanofilm is unstable and easily corroded, we controllably modified the graphene layer on the surface of the ACF layer by electrospraying, and this graphene modification could be carried out continuously on a meter scale. The modified graphene layer has no adverse effect on the light transmittance and conductivity of the ACF layer, but shows obvious corrosion resistance, and has a more obvious protective effect on ACF samples with high conductivity. After modification of the graphene layer, the corrosion rate of G/ACF can be reduced by 74.56% compared with that of the bare ACF sample. The conductivity of ACF samples without modification of graphene can be reduced by 34.1% after 72 h of salt spray test, while the conductivity of G/ACF samples with modification of graphene can be reduced by only 6.5%, which demonstrates the anti-corrosion ability of the graphene layer. Therefore, a new method with industrial potential to prepare robust large-area transparent conductive film is proposed. This method can also be extended to flexible substrates to prepare flexible transparent conductive films, which can be applied to the field of large-scale flexible transparent electronic devices.

## Figures and Tables

**Table 1:** Micrograph of ACF and G/ACF samples after salt spray test.

	0 h	24 h	48 h	72 h
Sample 1	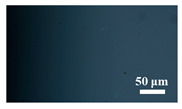	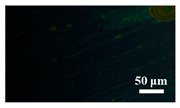	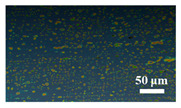	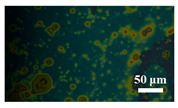
Sample 1G20	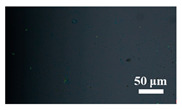	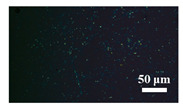	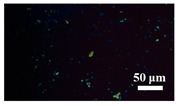	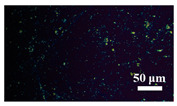
Sample 1G30	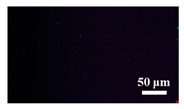	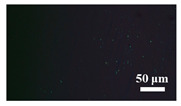	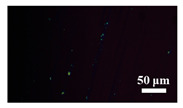	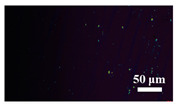
Sample 2	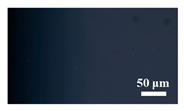	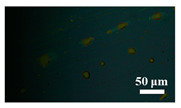	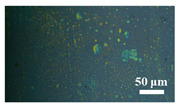	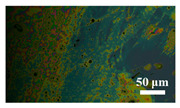
Sample 2G20	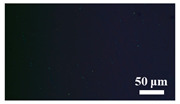	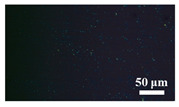	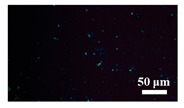	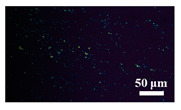
Sample 3G30	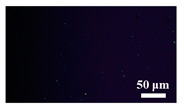	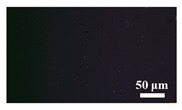	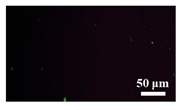	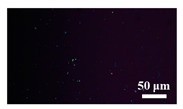

## Data Availability

Not applicable.
